# Uptake of ricinB-quantum dot nanoparticles by a macropinocytosis-like mechanism

**DOI:** 10.1186/1477-3155-10-33

**Published:** 2012-07-31

**Authors:** Tore Geir Iversen, Nadine Frerker, Kirsten Sandvig

**Affiliations:** 1 Centre for Cancer Biomedicine, Faculty Division Norwegian Radium Hospital, University of Oslo, Oslo, Norway; 2 Department of Biochemistry, Institute for Cancer Research, The Norwegian Radium Hospital, Oslo University Hospital, Montebello, Oslo, 0379, Norway; 3 Department of Molecular Biosciences, University of Oslo, Oslo, 0316, Norway

**Keywords:** Nanoparticles, Ligand binding, Diagnostic imaging, Endocytic mechanisms, Ricin, Dynamin

## Abstract

**Background:**

There is a huge effort in developing ligand-mediated targeting of nanoparticles to diseased cells and tissue. The plant toxin ricin has been shown to enter cells by utilizing both dynamin-dependent and -independent endocytic pathways. Thus, it is a representative ligand for addressing the important issue of whether even a relatively small ligand-nanoparticle conjugate can gain access to the same endocytic pathways as the free ligand.

**Results:**

Here we present a systematic study concerning the internalization mechanism of ricinB:Quantum dot (QD) nanoparticle conjugates in HeLa cells. Contrary to uptake of ricin itself, we found that internalization of ricinB:QDs was inhibited in HeLa cells expressing dominant-negative dynamin. Both clathrin-, Rho-dependent uptake as well as a specific form of macropinocytosis involve dynamin. However, the ricinB:QD uptake was not affected by siRNA-mediated knockdown of clathrin or inhibition of Rho-dependent uptake caused by treating cells with the *Clostridium* C3 transferase. RicinB:QD uptake was significantly reduced by cholesterol depletion with methyl-β-cyclodextrin and by inhibitors of actin polymerization such as cytochalasin D. Finally, we found that uptake of ricinB:QDs was blocked by the amiloride analog EIPA, an inhibitor of macropinocytosis. Upon entry, the ricinB:QDs co-localized with dextran, a marker for fluid-phase uptake. Thus, internalization of ricinB:QDs in HeLa cells critically relies on a dynamin-dependent macropinocytosis-like mechanism.

**Conclusions:**

Our results demonstrate that internalization of a ligand-nanoparticle conjugate can be dependent on other endocytic mechanisms than those used by the free ligand, highlighting the challenges of using ligand-mediated targeting of nanoparticles-based drug delivery vehicles to cells of diseased tissues.

## Background

Nanomedicine is an interdisciplinary field of research focusing on the development of nanoparticles (NPs) for clinical use in targeted drug delivery and diagnostic *in vivo* imaging. The goal will often be to increase the efficacy of drugs/ siRNAs at the target tissue and reduce the dose of drug into bystander tissue, and/or to develop NPs into diagnostic imaging agents specifically targeting tumors and diseased tissues. However, studies to fundamentally understand the mechanisms of cell-nanoparticle interactions are still lacking. Investigating whether the nanoparticles themselves might have adverse effects is also of crucial importance. The small sizes of nanoparticles enable them to cross various biological barriers of the body and also to enter the endocytic pathways of the cells, which in turn can give rise to unexpected toxicities. In a previous study, we demonstrated that cellular uptake of quantum dot (QD) nanocrystals that were surface modified with the targeting ligands transferrin (Tf) and ricin, perturbed normal intracellular trafficking in cells [1,2].

There are multiple types of endocytic pathways distinguished by specific molecular regulators. The clathrin-mediated endocytosis is by far the best studied of these mechanisms and was for a long time believed to be the only endocytic mechanism in addition to phagocytosis and macropinocytosis. However, several clathrin-independent mechanisms have been described, including dynamin-dependent mechanisms such as the RhoA- and caveolae-dependent, and dynamin-independent mechanisms such as the Cdc42-dependent and Arf6-dependent [3,4]. Dynamin is a large GTPase that mediates vesicle formation by its ability to tubulate and constrict membranes [5]. Caveolae-mediated uptake has been among the most studied routes of dynamin and cholesterol dependent endocytosis. In many studies uptake of nanoparticles (NPs) has been reported to occur via caveolae-mediated endocytosis merely based upon inhibited uptake by the pharmacological inhibitor methyl-β-cyclodextrin (mβCD). Notably, depleting the cell of cholesterol using mβCD also inhibits other endocytic mechanisms, such as clathrin-mediated endocytosis, phagocytosis and macropinocytosis [6,7]. Moreover, caveolae with a diameter of only 50–100 nm are clearly too small to be responsible for uptake of NPs larger than 100 nm. Caveolae are present in most vascular endothelia playing an important role in transcytosis of blood-borne molecules across the vascular endothelial cell layer, and transcytosis of 10–15 nm gold NPs linked with a caveolae-targeting ligand has been shown [8]. The belief that internalization via caveolae would spare its cargo from being degraded in lysosomes has also been a reason for ‘targeting’ NPs to caveolae. However, the previous model of caveolae giving rise to neutral “caveosomes” has now been revised: The caveosomes are artefacts obtained by overexpression of caveolin-1, and a ligand taken up by caveolae will enter endosomes and be transported to lysosomes [9].

Although, macropinocytosis in general has been considered to be a dynamin-independent mechanism, the ‘circular dorsal ruffle’-type of macropinocytosis might involve dynamin [10]. Macropinocytosis can in addition to fluid-phase uptake also accommodate uptake of particulate matter such as viruses, bacteria and nanoparticles [11,12]. Interestingly, dynamin-dependent and amiloride-sensitive macropinocytosis-like mechanisms have been reported for the uptake of bluetongue virus-1 and the Ebola virus [13,14]. In endothelial cells, multimeric antibody-nanoparticle conjugates directed against the intercellular adhesion molecule (ICAM-1) trigger internalization of large (diameter, 100–400 nm) anti-ICAM-1 and anti-PECAM-1 nanoconjugates by a macropinocytosis-like mechanism that is dynamin-dependent and also requires RhoA activation and actin reorganization [15,16]. In a recent study, it has been shown that endocytosis of chemokines in endothelial cell lines occurred via a macropinocytosis-like process that was not blocked by siRNA knock-down of PAK1 and CtBP1, two effector proteins of the Rho family GTPase Rac1 [17]. Furthermore, it has been found that specific splice-variants of dynamin-2 were required for the internalization of fluid by endocytic pathways distinct from macropinocytosis [18].

Toxins such as the plant toxin ricin have for many years been used as valuable tools to study intracellular transport routes in cells, and also uptake of ricin coupled to small gold NPs has been shown in Vero cells [19-22]. Ricin consists of two polypeptide moieties linked by a disulfide bond. The B-moiety binds to glycolipids and glycoproteins with terminal galactose, and can therefore be used as a membrane marker. Studies of ricin endocytosis after inhibition of clathrin-dependent endocytosis by different methods demonstrated that ricin was still endocytosed, and these studies provided some of the first evidence for clathrin-independent endocytosis [23,24]. Moreover, ricin was still endocytosed after overexpression of the dominant negative mutant dynamin (dyn K44A/G273D) [25], which inhibited clathrin-mediated endocytosis of both transferrin (Tf) and epidermal growth factor (EGF), whereas fluid phase uptake of horse raddish peroxidase was unaffected [26]. Thus, ricin can be internalized by clathrin and dynamin independent mechanisms in HeLa cells. These endocytic mechanisms still remain incompletely characterized, but recently some of the proteins involved have been identified (for review see [3]). In a previous study, we found the internalized ricin:QD NPs localizing to the same early and late endosomes as ricin itself, but in contrast to ricin which is also transported to the Golgi apparatus, Golgi transport of the ricin:QD conjugate could not be observed [1]. Recently, a few other studies also revealed a change in ligand behavior after conjugation to NPs: It has been demonstrated in pancreatic cancer cell lines that anti-EGFR antibody-gold nanoparticle conjugates used different and faster endocytosis mechanisms than the anti-EGFR antibody itself [27], and the valency of TatP domains conjugated to QDs affected the fate of the NPs [28]. Furthermore, multivalent binding of antibodies and ligands of the PECAM-1 glycoprotein to NPs has been shown to trigger internalization of the antibody-ligand nanoconjugates in endothelial cells although the antibodies themselves were not internalized [29].

In this study we investigated by which endocytic mechanisms small (30 nm) ricinB:QD NP conjugates were internalized, and identified a dynamin-dependent macropinocytosis-like mechanism to be critically involved.

## Results and discussion

Here, we have investigated the endocytic mechanisms responsible for internalization of ricinB:QDs, consisting of PEGylated streptavidin-coupled QDs that were conjugated with biotinylated ricinB (multivalent, molar ratio ricinB/QDs of 5), in HeLa cells. The size (hydrodynamic diam.) of the ricinB:QDs conjugates were measured by the Zetasizer to be 30 nm (5 nm variation between batches), small enough to be endocytosed by most endocytic mechanisms.

### Internalization of ricinB:QDs is mediated by dynamin-dependent endocytosis that is independent of clathrin

**Figure 1:**
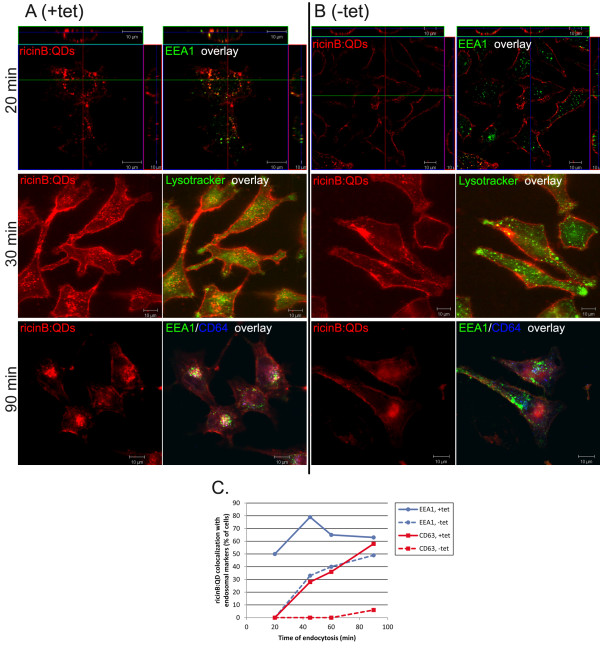
**Dynamin-dependent endocytosis of ricinB:QDs into HeLa cells.** Expression of the dominant-negative dynamin mutant was induced by removing tetracycline (B, -tet for 2 days), whereas control cells were grown in presence of tetracycline. All the cells were incubated with ricinB:QDs (100 nM: 20 nM) for various times at 37°C before fixation and fluorescent antibody labeling against EEA1 (green) and CD63 (blue), respectively. **A/B**: Internalization of ricinB:QDs into the HeLa, DynK44A cell line for 20 min, 30 min and 90 min. **C**: The kinetics of colocalization of ricinB:QDs and the endosomal markers EEA1(blue) and CD63 (red) in control cells (+tet) and in the mutant dynamin cells (−tet, dotted line), respectively. Quantification of the extent of colocalization between ricinB:QDs and EEA1 or CD63 (at each time point presented as percentage of cells displaying > 20% of total ricinB:QD pixels colocalizing with EEA1 and CD63, *n* = 30 cells pr. time point)

In our study of the endocytic mechanisms involved in uptake of ricinB:QD NP conjugates, we first investigated whether dynamin played a role. The role of dynamin-dependent endocytosis in uptake of ricinB:QDs was investigated in a HeLa cell line that expresses the dominant-negative dynamin-1 K44A mutant in a tetracycline-inducible manner [30]. Expression of the mutant dynamin K44A was induced by removal of tetracycline (−tetracycline) from the cells for 48 h prior to ricinB:QDs being added. Uptake of the fluorescent ricinB:QDs was analyzed by confocal microscopy (Figure 1). In control cells (A, +tetracycline), the ricinB:QDs were efficiently internalized within 20 min and found in endosomal ‘dot-like’ structures displaying a colocalization pattern with EEA1- and CD63-positive structures. In contrast, cells expressing the mutant dynamin K44A (B, -tetracycline) showed ricinB:QDs localized at the plasma membrane, and there was no significant colocalization with the endosomal markers. Furthermore, also during live-cell imaging of the HeLa cells, stained with the late endosomal (/low pH) marker LysoTracker, an inhibition in internalization of ricinB:QD and their colocalization with LysoTracker was observed (Figure 1B). However, after longer times of endocytosis, weak QD signals displaying colocalization with the endosomal markers (EEA1 and CD63) were also observed for the mutant dynamin expressing cells (−tet, 90 min, lower panel). Colocalization of ricinB:QDs with the early endosomal marker EEA1 and the lysosomal marker CD63 was quantified after increasing time of endocytosis into the cells (Figure 1C). In HeLa cells expressing mutant dynamin, no significant internalization of ricinB:QDs (measured as fraction of cells with EEA1 colocalization) was detected until 45 min of incubation. Moreover, even after 90 min of endocytosis in the mutant dynamin cells, a reduced uptake and transport of ricinB:QDs into EEA1 and CD63-positive endosomes was quantified. In contrast, the majority of control cells displayed up to 50% colocalization with the lysosomal marker CD63. The binding of ricinB:QDs was not significantly affected by the expression of dynamin K44A, and as expected the same cells displayed a strong inhibition of transferrin uptake due to a block in clathrin-mediated endocytosis (data not shown). Moreover, no significant binding or uptake of the unconjugated streptavidin-coated QDs was detected by confocal microscopy even after 4 h of incubation (negative control, not shown), confirming that conjugation of the QDs to biotinylated ricinB conferred specific binding and uptake into the cells. All together, our data show that dynamin is required for efficient uptake of the ricinB:QDs in HeLa cells. However, ricinB:QDs was also internalized slowly and inefficiently via a dynamin-independent mechanism. 

In earlier studies, it has been shown that overexpression of mutant dynamin induced other compensatory dynamin-independent endocytic mechanisms that acted with similar kinetics in uptake of cargo such as ricin and the fluid phase marker HRP [25,31]. However, the ricinB:QDs can obviously not be internalized efficiently via this compensatory mechanism.

**Figure 2:**
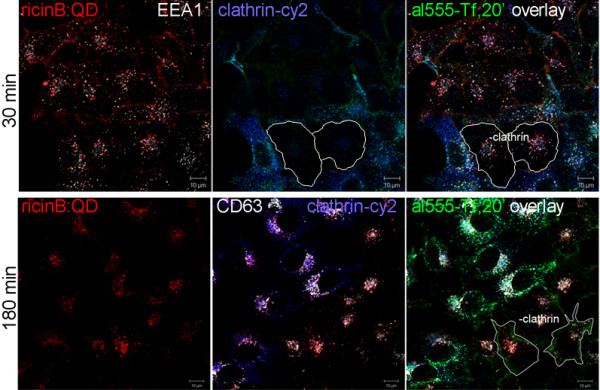
** Clathrin independent entry of the ricinB:QDs into HeLa cells.** HeLa cells were subjected to vector-based siRNA knock-down of clathrin heavy chain for 3 days. The ricinB:QD655 bioconjugates were allowed to be internalized into the cells at 37°C for 30 min and 180 min, respectively (representative images, upper/lower panel). Alexa555 (al555)-transferrin (Tf) (green) was added to cells for the last 20 min of the endocytosis period. The cells were then fixed and prepared for confocal immuno-fluorescence microscopy labeling them with antibodies against clathrin heavy-chain, EEA1 and CD63, and with the appropriate secondary antibody-Cy2 (blue)/Cy5 (white) fluorophore-conjugates. Images show normal uptake of ricinB:QD655 in cells were clathrin is knocked down (− clathrin, no blue staining), whereas the alexa555-Tf uptake was inhibited and only weakly stained the periphery of these cells.

The next step was to examine by which dynamin-dependent mechanism the ricinB:QD bioconjugate is endocytosed. First, we investigated the uptake in HeLa cells expressing vector-based clathrin siRNA (Figure 2). The transfected cells expressing clathrin siRNA did not stain positive for clathrin, and in these cells the uptake of fluorescently labeled transferrin was strongly inhibited (cells outlined in white). However, these clathrin siRNA cells did not display any significant reduction in uptake of the ricinB:QDs as compared to the untransfected cells. In control cells transfected with a plasmid expressing a scramble siRNA, no cells without clathrin expression and inhibited internalization of Tf were observed (data not shown). Moreover, a BHK cell line with inducible expression of clathrin antisense RNA [32] also showed normal uptake of ricinB:Qdot nanoconjugates (data not shown). 

### Cholesterol-dependent internalization of RicinB:QDs is independent of RhoA

**Figure 3:**
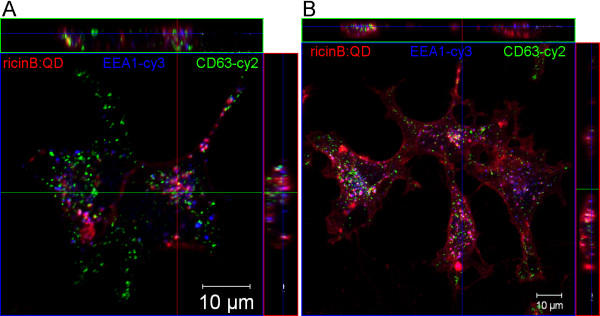
** Uptake of ricinB:QDs is not reduced by inhibition of Rho.(A)** Cells were left untreated, or **(B)** pre-treated with C3 transferase (1 μg/ml), followed by incubation with ricinB:QDs at 37°C for 40 min. Then, the cells were fixed and stained with the appropriate antibodies against the endosomal marker EEA1 (blue) and CD63 (green), respectively. Images include orthogonal views generated from a z-stack of images.

Because endocytosis of ricinB:QDs was critically dynamin-dependent, we further addressed whether an endocytic mechanism regulated by the small Rho-GTPases RhoA was involved [33]. The small GTPase RhoA also regulates formation of actin stress fibers and may be transiently activated during macropinocytosis [34]. To this end, we treated HeLa cells with the cell permeable C3 transferase, which selectively inhibits the small Rho-GTPases RhoA-B and –C [35]. HeLa Tet dynK44A cells were pre-treated with C3 transferase for 90 min or left untreated. Then, the cells were allowed to internalize the ricinB:QDs for 40 min before they were fixed for IF microscopy. The uptake of ricinB:QDs was shown by confocal imaging as they colocalized with endosomal markers and could be visualized inside the cells by z-stack imaging (Figure 3). Most of the ricinB:QDs was endocytosed within 40 min both in control cells and in the cells treated with C3 transferase, indicating that RhoA-dependent endocytic mechanisms did not play any significant role. Usually, inhibition of Rho-dependent endocytosis with the C3 transferase coincides with a change in morphology where the cells get smaller and less ‘spread out’. Here, we also observed a similar change in morphology of the treated cells (Figure 3B). Furthermore, in a recent study we found that the C3 transferase inhibited internalization of the *C. botulinum* C2 toxin in these HeLa cells [36]. 

**Figure 4:**
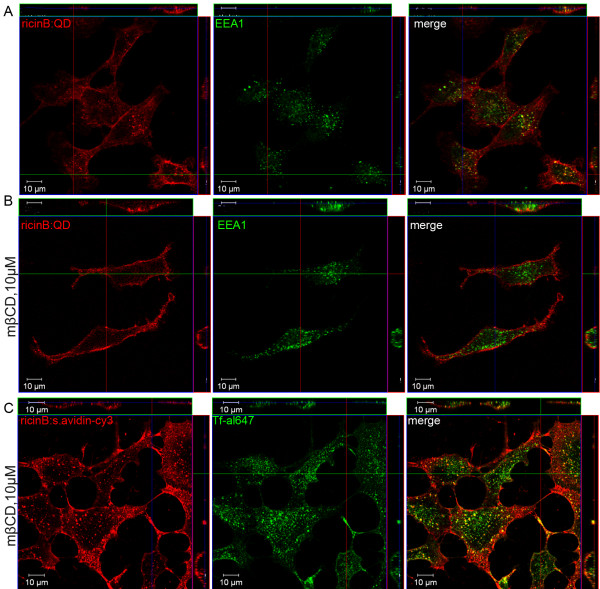
** Inhibited uptake of ricinB:QDs upon cholesterol depletion.** The HeLa cells were left untreated **(A)** or were pretreated with methyl-β-cyclodextrin (mβCD, 10 μM) for 30 min (**B** and **C**). Then, the cells were allowed to bind and endocytose ricinB, ricinB:QDs (red), or Tf-alexa647 (green) for 30 min. The cells were fixed and stained with an antibody against EEA1 followed by the appropriate secondary ab-Cy2 (green) conjugate or with streptavidin-Cy3 (red) conjugate to stain for the biotinylated ricinB.

Cholesterol is a critical constituent of lipid rafts and has been reported to play an important role in various mechanisms of endocytosis such as clathrin-mediated, caveolae-mediated, flotillin-mediated and macropinocytosis [3,7]. Therefore, we decided to deplete the HeLa cells for cholesterol by pretreatment for 30 min with the inhibitor mβCD (10 μM). The cells were then allowed to internalize ricinB:QDs (Figure 4A,B) or ricinB-biotin:streptavidin-Cy3 conjugates together with Tf (Figure 4C), respectively for 30 min. Figure 4B shows that the uptake of ricinB:QDs was strongly reduced in cells depleted of cholesterol, since no colocalization with the endosomal marker EEA1 was observed and side views of the cells revealed ricinB:QD staining of the cell surface only. This result shows the involvement of cholesterol in uptake of the ricinB:QDs. Notably, caveolae-mediated endocytosis is not likely to play any important role in the uptake of ricinB:QDs, because the HeLa dynK44A cells contain very few caveolae [37]. In a previous study, we demonstrated that cholesterol depletion by mβCD in HeLa cells only reduced endocytosis of Tf by 50% whereas ricin uptake was reduced by < 20% [6]. Thus, as a positive control we show that the cells treated with mβCD still internalized ricinB and Tf (Figure 4C), as fluorescently labeled ricinB (red color) and Tf (green color) displayed significant colocalization in endosomal ‘dots’ within the cells. 

### Effects of actin inhibitors on the ricinB:QD endocytosis

**Figure 5:**
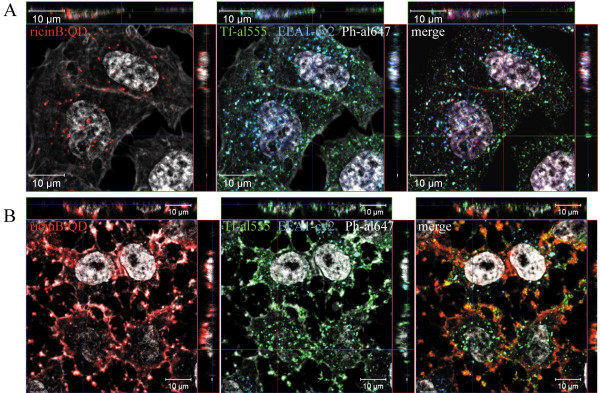
** Actin-dependent uptake of the ricinB:QDs.** HeLa cells were left untreated **(A)** or treated with cytD **(B)**. Then, ricinB:QDs (in red) and transferrin- alexa555 (Tf-al555, green) were endocytosed for 30 min at 37°C. The cells were fixed and stained with an anti-EEA1 antibody and the appropriate secondary antibody-Cy2 conjugate (blue) together with phalloidin (Ph)-alexa647 (white) for staining of actin. The cell nuclei were stained with Hoechst33342 (white).

In order to address the role of actin filament assembly in uptake of the ricinB:QDs, HeLa cells were pre-treated with the inhibitor cytochalasin D (cytD, 5 μM). CytD is a cell-membrane permeant that caps actin filaments thereby preventing further polymerization and ultimately resulting in actin filament disassembly. As seen in Figure 5B, uptake of the ricinB:QDs was strongly reduced by cytD, as most ricinB:QDs were localized along the cell periphery. Uptake of Tf-al555 was not significantly inhibited by cytD and displayed colocalization with EEA1 (blue) in endosomal ‘spots’.

In contrast to our findings, endocytosis of small particles such as QDs (<100 nm) have been reported to be insensitive to cytD in mammalian macrophages, leukocytes and dendritic cells [38,39]. However, disruption of F actin by cytD has been reported to play a variable role in endocytosis of Tf depending on both the cell line and whether the cells were grown in suspension or not [40,41]. Recently, is has been shown that membrane tension can decide whether actin is required for clathrin-dependent endocytosis [41].

### Internalization of ricinB:QDs is mediated by a macropinocytosis-like mechanism

**Figure 6:**
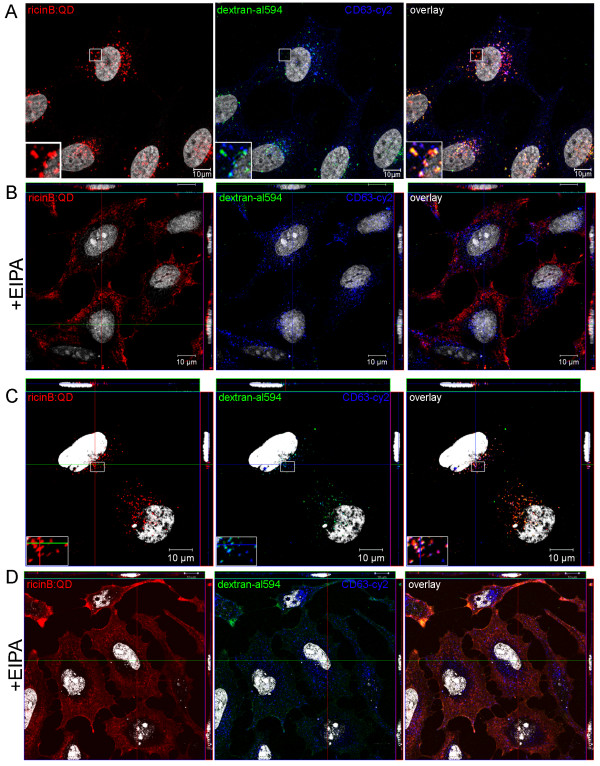
** Inhibition of ricinB:QDot endocytosis upon treatment of cells with EIPA, an inhibitor of macropinocytosis.** RicinB: QDs (red) were pre-bound to HeLa cells at 4°C, and then internalized in the presence of alexa594 dextran (green) for 30 min (panel A,B) and 90 min (panel C,D), respectively. The cells were fixed and stained with lysosomal antibody CD63 and its corresponding secondary antibody-Cy2 (blue). The cell nuclei were stained with Hoechst33342 (white).

Macropinocytosis is known to be critically dependent on the actin cytoskeleton for generating the plasma membrane ‘ruffles’ or filopodia that fold back on the plasma membrane to form the macropinosomes. Macropinocytosis is dependent upon the amiloride-sensitive Na^+^/H^+^ exchanger in the plasma membrane and is inhibited by 5-(N-ethyl-N-isopropyl)-amiloride (EIPA). In a recent study it has been shown that the inhibitory effects of amiloride and related compounds are the consequence of submembranous acidification caused by H^+^ generation and the blocked Na^+^/H^+^ exchange across the plasma membrane [42]. The macropinosomes that pinches off from the cell surface can be identified through the use of fluid phase markers such as dextran, horse radish peroxidase and Lucifer Yellow. Upon co-internalization of ricinB:QDs and dextran, we observed good co-localization between them within endosomal structures (yellow color) even after short times of internalization (< 30 min., Figure 6A), indicating that they were endocytosed by the same mechanism. To ascertain that this colocalization was not an artifact due to cross-talk of the QD signal into the dextran-alexa594 channel, we showed that the dextran signal within the rectangle area (Figure 6A, inset shows pre-bleech) could be photo-bleached using high laser power whereas the QD signal persisted. Furthermore, we observed an increasing colocalization between ricinB:QDs and the lysosomal marker CD63 from 30 min to 90 min (shown as purple color, Figure 6A,C insets), indicative of routing of the ricinB:QDs into lysosomes/late endosomes. Importantly, Figure 6 (B and D) shows that EIPA efficiently inhibited uptake of the ricinB:QDs and dextran in HeLa cells. In the EIPA-treated cells, the ricinB: QDs were still retained at the cell surface after 90 min of endocytosis. In contrast, EIPA did not inhibit uptake of transferrin as most EIPA-treated cells showed Tf uptake (not shown). 

The activation of PI3K and the engagement of signaling molecules including Rac1, Arf6 and the RhoA GTPase are common to a variety of actin-dependent processes such as phagocytosis and macropinocytosis [43]. In this context, it is interesting to note that in this study uptake of ricinB: QDs were not inhibited by treatment of the HeLa cells with the inhibitor of Rho, C3 transferase (see Figure 3). Furthermore, overexpression of the dominant-negative mutant Arf6 T27N in a stably transfected HeLa cell line [44] did not inhibit endocytosis of ricinB: QDs (data not shown). Macropinocytosis has been considered as a regulated form of endocytosis induced in response to growth factors such as epidermal growth factor [45]. Therefore, we would like to know whether the ricinB: QDs could induce such a fluid phase pathway. Using the Olympus ScanR fluorescence microscope, we quantified the uptake of fluorescent dextran-alexa594 in HeLa cells when taken up alone or when co-internalized with the ricinB:QDs for 30 min. No differences in the fluorescence intensities of internalized dextran were measured between the two conditions (data not shown), indicating that uptake of the ricinB:QDs did not significantly change the fluid uptake of dextran during this time period.

## Conclusions

**Figure 7:**
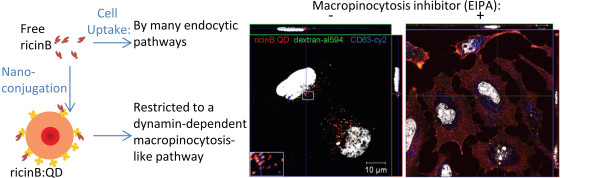
** Endocytosis of ricinB:QD nanocomplexes is restricted to a macropinocytosis-like pathway.** Ricin:QD NPs cannot exploit the various endocytic pathways accessible to ricin toxin itself.

In this study we investigated the relevance of various endocytic pathways in the uptake of ricin-coupled QD nanoparticles conjugates in HeLa cells. We demonstrated that the ricin-QDs conjugates were endocytosed by a dynamin-dependent but clathrin-independent pathway. Furthermore, treatment of the cells with C3 transferase to inhibit the Rho-mediated endocytic mechanism did not affect ricinB:QD uptake. Depletion of cholesterol from the cells, significantly reduced the uptake of ricinB:QDs. Interestingly, treatment of the cells with an inhibitor of macropinocytosis, the amiloride-analog EIPA, strongly reduced the uptake of ricinB: QDs. Thus, we have demonstrated that a dynamin-dependent and macropinocytosis-like pathway is necessary for the efficient endocytosis of ricinB:QD nanoconjugates. In contrast, ricin alone is internalized equally efficiently both by dynamin-dependent and dynamin-independent endocytic pathways. These endocytic pathways seem to be unavailable for internalization of the ricinB:QDs, and their uptake is instead triggered via a specific endocytic pathway (Figure 7). These results demonstrate that one needs to be cautious when ligands are coupled to nanoparticles with the intention of achieving efficient NP delivery into certain cells or tissues by a given endocytic mechanism. Clearly, the endocytic pathways operating after nanoconjugation warrants further investigations. The size of the NPs to which the ligands are conjugated is certainly also an issue, as larger NPs cannot enter into some endocytic pathways due to sterical hindrance. Importantly, the multivalency of ligand-nanoparticle conjugates might not only increase the affinity for a cell, but also enable more efficient receptor clustering resulting in induction of signaling and thereby triggering of alternative endocytic pathways.

## Methods

### Experimental reagents

Reagents used in this study included the amiloride analoge EIPA (Sigma), cytochalasin D (Sigma), methyl-β-cyclodextrin (Sigma), ADP-ribosyltranferase C3 (Sigma). RicinB subunit was purchased from Vector Laboratories (Burlingame, CA). Quantum dots 655 nm, alexa-555 transferrin (Tf), alexa-594 dextran, alexa-647 phalloidin (Ph), and Hoechst 33342 were purchased from Invitrogen (Carlsbad, CA). The following antibodies were used: Rabbit anti-EEA1 (Cell Signaling Technologies), mouse anti-CD63 (Developmental Studies Hybridoma Bank, Univ. of Iowa). The Cy2- and Cy3-labeled secondary antibody conjugates of donkey anti-rabbit and donkey anti-mouse were purchased from Jackson ImmunoResearch Laboratories (West Grove, PA, USA).

### Biotinylation of ricinB

RicinB (1 mg/ml) was biotinylated according to manufacturer’s instructions using NHS-SS-biotin (Pierce, Rockford, IL) in a 1:20 molar ratio of ricin:biotin. The reaction took place at room temperature in the dark for 45 min. Unincorporated NHS-SS-biotin was removed using a spin column (Micron, YM-10, Millipore).

### Hydrodynamic size measurement

The hydrodynamic diameter of the streptavidin-coupled QD655 particles was measured in PBS buffer or in cell culture medium using using a Zetasizer Nano ZS (Malvern Instruments Ltd, Worcestershire, UK).

### Cell culture

HeLa cells were maintained at 37°C and 5% CO_2_ in Dulbecco’s Modified Eagle medium, DMEM (Invitrogen, Carlsbad, CA, USA), supplemented with 10% v/v fetal bovine serum (PAA Laboratories, Linz, Austria), 100 U/ml penicillin (Invitrogen) and 100 μg/ml streptomycin (Invitrogen).

### Cellular uptake of ricinB:qdot nanoconjugates by confocal fluorescence microscopy

HeLa cells or HeLa Dyn-K44A cells (seeded 2x10^4^cells/well in 24-well trays) were cultured on coverslips for 24 h or 48 h prior to the experiments, respectively. Then, ricinB:QDs conjugates were prepared directly at the cell surface, as previously described:[1] Cells were washed in cold Hepes-medium prior to incubation with the biotinylated ricinB (200 nM) ligand in Hepes medium for 10 min on ice. The cells were briefly washed 2x in tetra-borate buffer (50 mM sodium-borate, pH8.3; 215 mM sucrose) and then incubated with the streptavidin-coupled QD655 (20 nM) in tetra-borate buffer for 5 min on ice [46]. Subsequently, the cells were washed 2x in Hepes medium before endocytosis of the ricinB:QDs (molar ratio, 5:1) was performed by incubating the cells in Hepes medium at 37C for various times with or without the appropriate inhibitors. The ricinB:QDs were also co-internalized with other ligands such as Tf-alexa-555 and dextran-alexa-647.

After fixation in 10% (w/v) formalin for 15 min, the cells were permeabilized and blocked in 0.1% Triton X-100 and 1% BSA in PBS for 1 hour at room temperature. The cells were immuno-stained with the following primary antibodies: mouse anti-EEA1 ab. (1:100) and mouse anti-CD63 ab. (1:200). The secondary antibody-fluorescent dye conjugates used were: Donkey anti-rabbit-Cy2 (1:200), donkey anti-rabbit-Cy3 (1:500) and donkey anti-mouse-Cy2 (1:500). Coverslips were mounted in Mowiol (Calbiochem) and examined using a confocal microscope (LSM 788; Carl Zeiss MicroImaging, Inc.) equipped with a Neo-Fluar 63x/1.45 oil immersion objective. Image processing and analysis were done with Zeiss LSM 510 software version 3.2 and Adobe Photoshop 7.0.

### Vector-based siRNA knock-down of clathrin

Clathrin heavy-chain was knocked down with a vector-based siRNA construct [47]. Cells were transiently transfected for 3 days with plasmid DNA using Fugene 6 (Roche Diagnostics, Mannheim, Germany), according to the manufacturer’s protocol.

## Competing interests

The authors declare that they have no competing interests.

## Authors’ contributions

TGI conceived the idea of this study and participated in its design, carried out most of the experiments on endocytosis mechanisms and drafted the manuscript. NF carried out quantitative colocalization analysis of confocal images using the Zeiss lsm 510 software (version 3.2), and performed the cellular uptake study after cholesterol depletion using methyl-β-cyclodextrin. KS participated in study design and coordination and helped draft the manuscript. All authors read and approved the final manuscript.
